# The immunomodulatory quinoline-3-carboxamide paquinimod reverses established fibrosis in a novel mouse model for liver fibrosis

**DOI:** 10.1371/journal.pone.0203228

**Published:** 2018-09-05

**Authors:** Nina Fransén Pettersson, Adnan Deronic, Julia Nilsson, Tine D. Hannibal, Lisbeth Hansen, Anja Schmidt-Christensen, Fredrik Ivars, Dan Holmberg

**Affiliations:** Department of Experimental Medical Science, Lund University, Lund, Sweden; University of Navarra School of Medicine and Center for Applied Medical Research (CIMA), SPAIN

## Abstract

Quinoline-3-carboxamides (Q substances) are small molecule compounds with anti-inflammatory properties. In this study, we used one of these substances, Paquinimod, to treat a novel model for chronic liver inflammation and liver fibrosis, the NOD-Inflammation Fibrosis (N-IF) mouse. We show that treatment of N-IF mice significantly reduced inflammation and resulted in the regression of fibrosis, even when the treatment was initiated after onset of disease. The reduced disease phenotype was associated with a systemic decrease in the number and reduced activation of disease-promoting transgenic natural killer T (NKT)-II cells and their type 2-cytokine expression profile. Paquinimod treatment also led to a reduction of CD115^+^ Ly6C^hi^ monocytes and CD11b^+^ F4/80^+^ CD206^+^ macrophages.

## Introduction

The quinoline-3-carboxamides (Q compounds) are small molecule compounds that have exhibited beneficial effects in several mouse models of inflammatory disease. One such compound, Paquinimod (ABR-215757), has been shown to ameliorate collagenase-induced osteoarthritis [1] as well as symptoms in mouse models of multiple sclerosis and experimental autoimmune encephalomyelitis (EAE) [2]. Phas also had beneficial effects in systemic lupus erythematosus (SLE) patients [3] and, more recently, in systemic sclerosis (SSc) patients [4]. Q compounds, such as Paquinimod have been reported to bind to the protein S100A9 [5], a pro-inflammatory mediator that also possesses chemotactic effects and is involved in the recruitment of cells of myeloid origin [6, 7]. The interaction of S100A9 with its receptors is inhibited by Paquinimod [5], and this inhibition has been suggested to underlie the anti-inflammatory effects of Paquinimod [2].

In this study, we evaluated the efficacy of Paquinimod in inhibiting the spontaneous, chronic liver inflammation and fibrosis that occur in the recently established NOD-Inflammation Fibrosis (N-IF) mouse model [8]. N-IF mice spontaneously develop chronic inflammation and liver fibrosis driven by T-cell receptor (TCR) transgenic natural killer T (NKT)-II cells generated in immunodeficient NOD.Rag2^-/-^ mice. Several components of the liver pathology in the N-IF mouse overlap with those of human conditions in which a progressive chronic inflammation precedes the development of fibrosis. Moreover, the fibrosis developing in the N-IF mouse liver has been demonstrated to be associated with an accumulation of α-SMA expressing hepatic stellate cells [8]. These phenotypic similarities make the N-IF mouse model unique compared to other available rodent models for liver fibrosis and provides a novel tool to test the efficacy of anti-fibrotic drug candidates.

Using this model, we here show that Paquinimod has a therapeutic effect not only on the inflammation in the N-IF mouse liver but also significantly reduces the established liver fibrosis. This was accompanied with reduced numbers of TCR transgenic NKT-II cells, and their type 2-cytokine expression, as well as with reduced numbers of CD115^+^ Ly6C^hi^ monocytes and CD11b^+^ F4/80^+^ CD206^+^ macrophages.

## Material and methods

### Ethics statement

Animal experiments were performed in strict accordance with the recommendations for the use of laboratory animals from the Swedish Board of Agriculture. The ethics committees of the local committee on the ethics of animal experiments of Malmö and Lund permits no M143-11 and M52-16. Mice were sacrificed by cervical dislocation. All efforts were made to minimize suffering.

### Animal studies

N-IF (24αβNOD.Rag2^-/-^) and 24αβNOD control mice were bred and maintained in a specific pathogen-free facility at the Biomedical Center animal facility in Lund, Sweden. To study the effects of the Q compound Paquinimod, mice aged 8–9 weeks were treated with Paquinimod dissolved in drinking water corresponding to a daily dose of 25 mg/kg body weight/day for 4 or 10 weeks. Paquinimod was provided by Active Biotech, Lund, Sweden.

### Histology

Following 4 or 10 weeks of treatment, the mice were anesthetized and perfused with PBS via intra-cardiac puncture. Livers and spleens were weighed, and organ biopsies were fixed in 4% neutral-buffered formalin, embedded in paraffin and sectioned. Sections (5 μm) were stained with hematoxylin and eosin (H&E) and Masson’s trichrome and evaluated by microscopy.

### Hydroxyproline measurement

The hydroxyproline content of the liver and spleen was determined using the Hydroxyproline Colorimetric Assay Kit (BioVision, Milpitas, CA, USA) according to manufacturer’s instructions. Briefly, 100 μl of wet tissue (10 mg) was hydrolyzed in 100 μl of 12 N HCl for 3 h at 120°C. Aliquots were transferred to a 96-well plate, dried overnight and incubated with chloramine T and DMAB reagents for 90 min at 60°C. Finally, the absorbance at 560 nm was measured.

### Gene expression analysis

RNA was extracted from liver and spleen biopsies by the homogenization of tissues in QIAzol Lysis Reagent (Qiagen, Hilden, Germany), followed by purification on RNeasy Mini columns (Qiagen) according to the manufacturer’s protocol. RNA quality was evaluated using the A_280_/A_260_ ratio. One μg of total RNA was reverse transcribed using the RT^2^ HT First Strand Kit (Qiagen). Next, cDNA synthesis was performed in a C1000 Thermal Cycler (Bio-Rad, Hercules, CA) at 42°C for 15 min, and the reaction was terminated at 95°C for 5 min. The cDNA was subsequently used for quantitative PCR analysis using the RT^2^ SYBR Green MasterMix (Qiagen) at 95°C for 5 min, followed by 40 cycles at 95°C for 15 sec and 60°C for 1 min in a MyiQ Thermal Cycler (Bio-Rad). The analyses were performed using primers for mouse CCR2, MMP2, MMP8 and TGF-β. GAPDH was used as a housekeeping gene. Fold changes in mRNA expression of the genes were calculated by using the ΔΔCt method. The relative normalized expression of each gene for each sample was calculated using the formula 2^-ΔΔCt^ where the 24αβNOD mice treated with vehicle for 4 weeks is the control group.

### Cell preparation

Leukocytes from livers were obtained by incubating liver sections in a solution of 1 mg/ml collagenase XI (Sigma-Aldrich, St. Louis, MO) for 40 min at 37°C, mincing the tissue through a 70-μm mesh and separating the leukocytes by centrifugation with a 50/30 Percoll gradient (GE Healthcare, Uppsala, Sweden). Single-cell suspensions from the spleen were prepared by disrupting the tissue through a 70-μm mesh.

### Antibodies and flow cytometry

Streptavidin-Brilliant Violet 605 and the following antibodies were purchased from Biolegend (Nordic Biosite, Täby, Sweden): anti-c-kit-APC-Cy7 (2B8), anti-CD11b-Alexa700 (M1/70), anti-CD45-APC-Cy7 (30-F11), anti-CD115-APC (AFS98), anti-CD206-FITC (C068C2), anti-F4/80-PE-Cy7 (BM8), and anti-Ly6G-Brilliant Violet 421 (1A8). The following antibodies were purchased from eBioscience (Nordic Biosite): anti-CD11c-PE-Cy7 (N418), anti-FcεR1-APC (MAR-1), anti-Vα3.2-APC (RR3-16), and anti-Vβ9-eFluor450 (MR10-2). Ly6C-biotin (AL-21) and SiglecF-PE (E50-2440) were purchased from BD Biosciences (Stockholm, Sweden), goat anti-S100A9 (mAb. Santa Cruz, sc-8115, clone M-19) was purchased from Santa Cruz Biotechnology, Dallas, Texas). Prior to surface staining, cells were incubated with the 2.4G2 (anti-CD16/CD32) antibody (BD Biosciences) to prevent nonspecific binding. The cells were then stained with the surface antibodies in FACS buffer (PBS supplemented with 3% FCS). Fixable Viability Dye-eFluor506 purchased from eBioscience was used to detect dead cells. For intracellular staining of CD206, the Foxp3/Transcription Factor Staining Kit from eBioscience was used according to the manufacturer’s protocol. The analysis of stained cells was performed using the LSRII flow cytometer (BD Biosciences) and FlowJo software (TreeStar, Ashland, OR). For gating strategy see S1 Fig.

### Cell activation and cytokine analysis

Single cells from the spleen or liver (10^5^ per well) were cultured in complete medium [RPMI 1640 medium supplemented with 10% FCS, 100 U/ml penicillin/streptomycin, 2.5% sodium bicarbonate (7.5% solution), 1 mM sodium pyruvate and 69 μM 1-thioglycerol] and activated with anti-CD3 (4 μg/ml, clone 154-2C11, BD Biosciences) antibody. The supernatants were collected after 24 h and were analyzed for cytokines using the mouse Th1/Th2/Th17/Th22 13-plex (eBioscience) according to the manufacturer’s instructions.

### Statistical analysis

The results are presented as the means and standard error of the mean (SEM). Differences between two groups were considered significant when P<0.05, as assessed using Mann-Whitney nonparametric two tailed rank test (alpha = 0.05) by GraphPad Prism 5 software, San Diego, CA).

## Results

### Paquinimod reduces inflammation in N-IF mice

**Fig 1:**
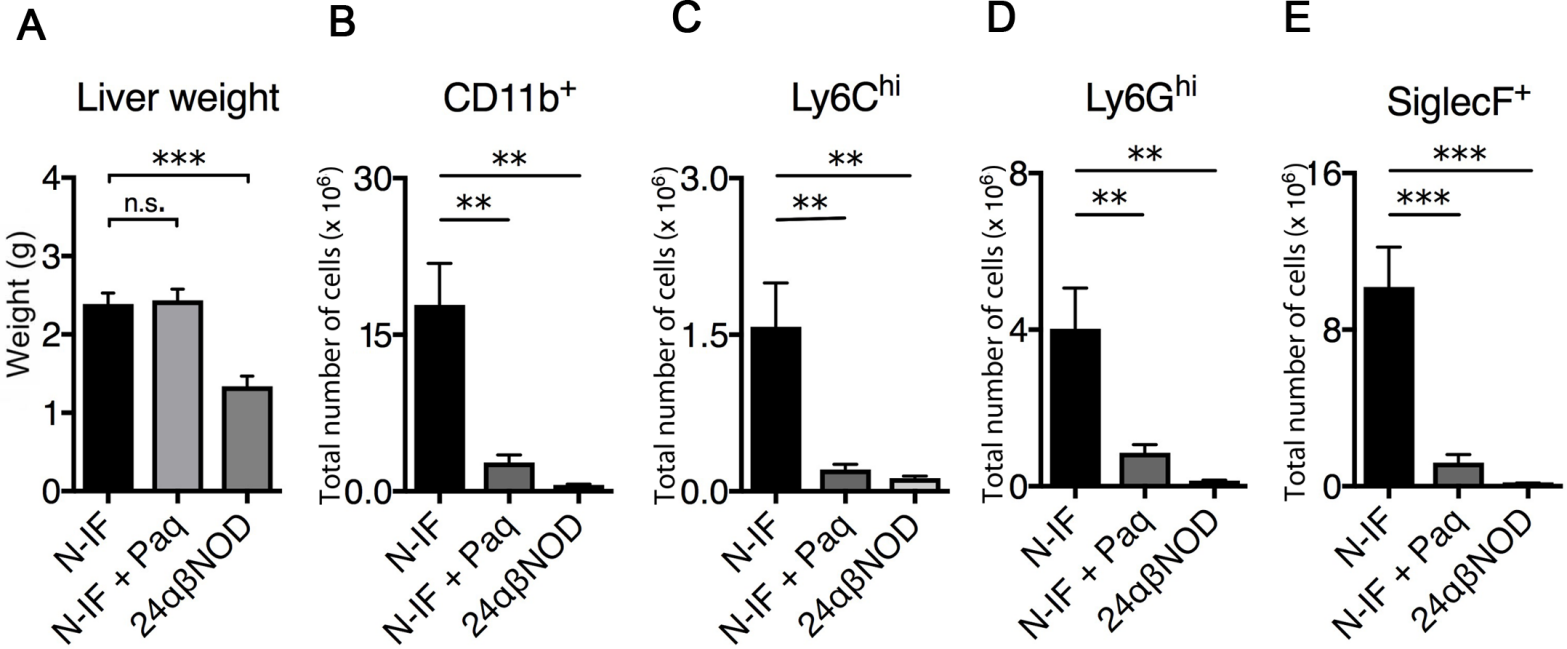
Paquinimod reduces accumulation of inflammatory cells in the liver of N-IF mice. (A) Liver weights from non-treated, 12 weeks old N-IF mice (n = 4), N-IF mice treated with Paquinimod for 4 weeks and sacrificed at 12 weeks (n = 6), and 12 weeks old non-treated 24αβNOD mice (n = 5). (B-E) Single cell suspensions from liver analyzed by flow cytometry. Total number of (B) CD11b^+^ cells gated from viable CD45^+^ cells, and (C) of Ly6C^hi^, (D) Ly6G^hi^ and (E) SiglecF^+^ cells gated from CD11b^+^ cells. Representative results of two independent experiments are shown. *P<0.05, **P<0.01, ***P<0.001, ****P<0.0001, unpaired **t**-test.

**Fig 2:**
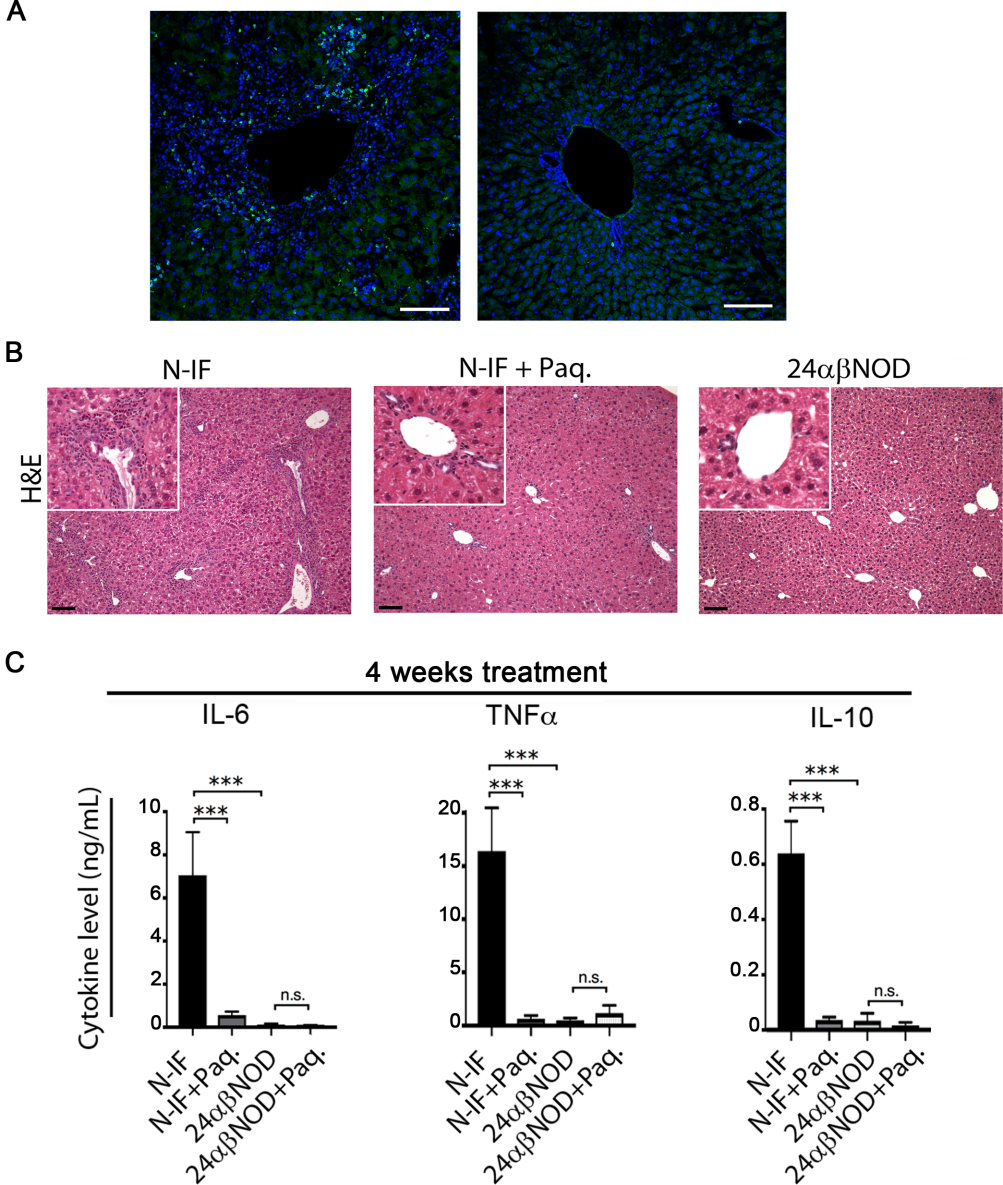
Paquinimod reduces the inflammation in N-IF mice. (A) Immunohistochemical staining of representative liver sections from 8 weeks old N-IF mice (left) and 24αβNOD control mice (right) showing anti-S100A9 (green) and DAPI (blue). scale bar: 100μm. (B) Representative H&E-stained liver sections from non-treated N-IF mice (N-IF), N-IF mice treated with Paquinimod for 4 weeks (N-IF+Paq.) and non-treated 24αβNOD control mice (24αβNOD). Scale bars are 100 μm. (C) TNFα, IL-6 and IL-10 levels in supernatants total liver leukocytes from non-treated N-IF mice (n = 10), N-IF mice treated with Paquinimod for 4 weeks (n = 14), non-treated 24αβNOD mice (n = 13) and 24αβNOD mice treated with Paquinimod for 4 weeks (n = 12). Isolated cells were cultured for 24 h with anti-CD3 (4 μg/ml) activation. The results are from three pooled experiments. n.s. = not significant; *P<0.05, **P<0.01, ****P<0.0001, unpaired *t*-test.

The N-IF mouse model spontaneously develops chronic inflammation and fibrosis in multiple organs and tissues, including in the liver [8]. In accordance with this, 8-9-week-old N-IF mice have an increased liver weight (Fig 1A) with accumulation of CD45^+^ cells compared to control 24αβNOD mice (Fig 1B–1E). CD11b^+^ myeloid cells predominated by CD11b^+^SiglecF^+^ eosinophils constituted a major cellular component of the inflammatory cells that accumulate in the liver of N-IF mice (Fig 1B–1E). Previous studies using Paquinimod or other quinoline-3-carboxamides have shown that such compounds reduce the accumulation of myeloid cell populations such as inflammatory monocytes and eosinophils at sites of acute inflammation [2]. Because Paquinimod has been reported to bind to the protein S100A9 [5] and to be involved in the recruitment of cells of myeloid origin [6, 7], we next confirmed that cells expressing S100A9 were accumulating in the liver of N-IF mice (Fig 2A). To evaluate whether a similar effect of Paquinimod could be observed in N-IF mice, the substance was administered via the drinking water to 8-9-week-old mice for a period of 4 weeks. This treatment regimen led to a significant reduction in the accumulation of CD11b^+^ cells in the liver (Fig 1B). Consistent with these results, the numbers of the major CD11b^+^ subpopulations; monocytes (CD11b^+^Ly6C^hi^) (Fig 1C), neutrophils (CD11b^+^Ly6G^hi^) (Fig 1D) and eosinophils (CD11b^+^SiglecF^+^) (Fig 1E), were significantly reduced in Paquinimod-treated N-IF mice.

In addition to the overall reduction in CD11b^+^ cells, we also noted an alteration in the composition of the major CD11b^+^ subpopulations. Thus, Paquinimod treatment led to an increased frequency of Ly6G^hi^ neutrophils and a reduction in the frequency of SiglecF^+^ eosinophils in the N-IF mouse liver (S2 Fig). Histological analysis confirmed the accumulation of inflammatory cells in the untreated N-IF mouse liver and a significant reduction in infiltrating inflammatory cells in the liver upon 4 weeks of Paquinimod treatment (Fig 2). In line with this, we also noted a reduction in the expression of both pro and anti-inflammatory cytokines such as TNFα, IL-6 and IL-10 after 4 weeks treatment (Fig 2C) as well as after 10 weeks treatment (S3 Fig).

### Paquinimod reduces fibrosis in N-IF mice

**Fig 3:**
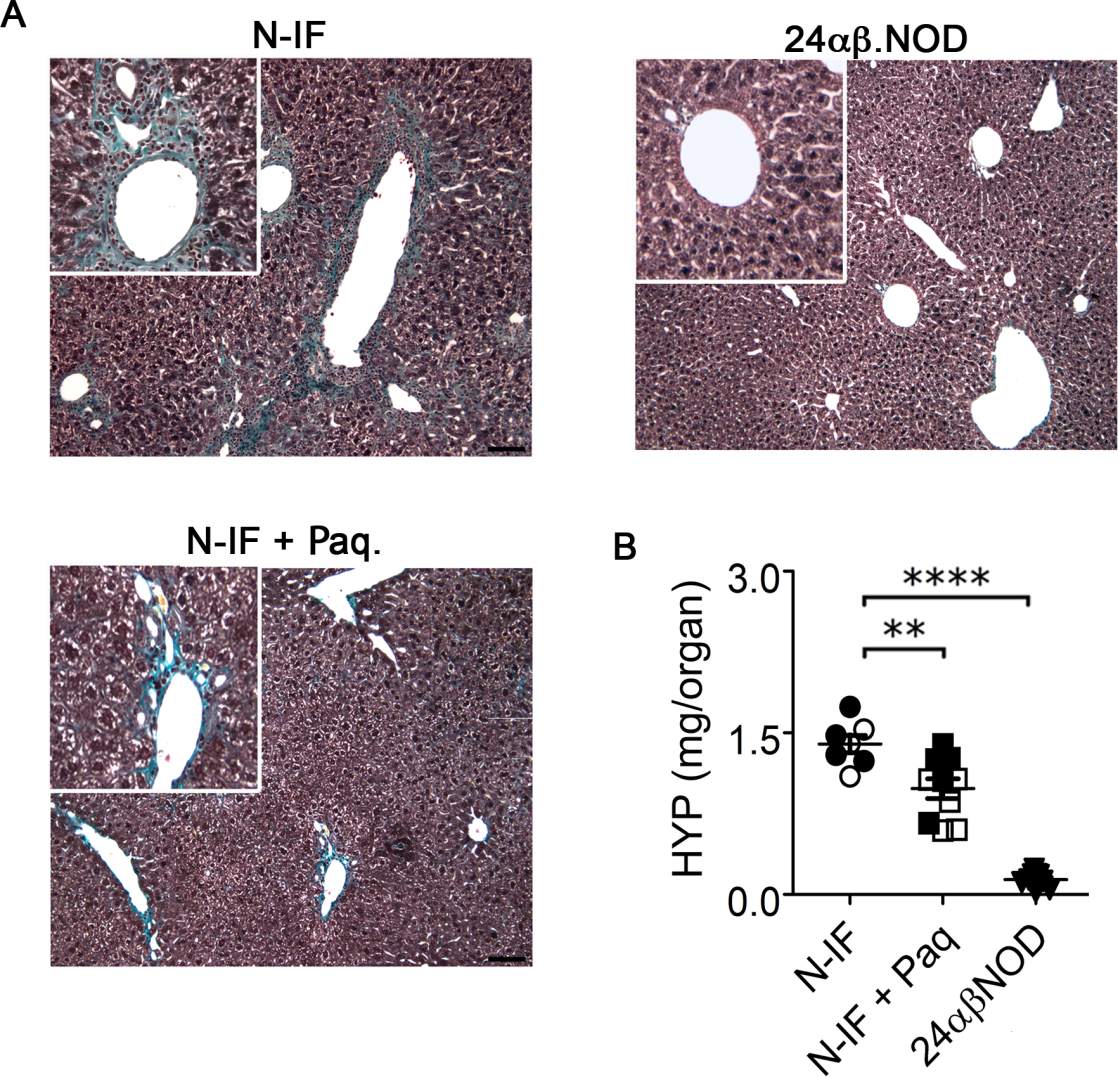
Paquinimod reduces liver fibrosis in N-IF mice. (A) Representative Masson’s trichrome-stained sections of liver from non-treated N-IF mice (n = 7), N-IF mice treated with Paquinimod for 4 weeks (n = 10) and non-treated 24αβNOD control mice (n = 9). (B) Quantification of collagen in the liver based on hydroxyproline measurements. Representative results of two independent experiments are shown.

The N-IF mouse develops extensive liver fibrosis in the liver primarily localized to the portal tracts and central veins with some periportal and bridging fibrosis [8] and Fig 3. Since Paquinimod treatment had a potent anti-inflammatory effect in this model, Histological analysis of liver sections stained with Masson’s trichrome staining revealed that Paquinimod treatment for 4 weeks significantly reduced liver fibrosis in the N-IF mouse (Fig 3A). A similar effect was observed when treatment was extended to 10 weeks (S4 Fig). To quantify this effect of Paquinimod treatment on fibrosis, we determined the levels of tissue hydroxyproline, a major component of collagen. This analysis confirmed the observations from the histological analyses and revealed a reduction in the high levels of hydroxyproline in the liver of Paquinimod treated N-IF mice (Fig 3B). Because the 8-week-old N-IF mice already displayed areas of collagen deposits in the liver at the onset of treatment, these data suggest that Paquinimod treatment reduces the already established fibrosis in the N-IF mice.

In contrast to the pronounced effect of Paquinimod on the liver inflammation and fibrosis in the N-IF mouse, we noted no reduction in liver weight in the treated animals (Fig 1A). Because Q compounds are metabolized in the liver and have been reported to lead to activation of hepatocytes [9] we reasoned that the lack of reduction in liver weight could be due to this metabolic activation in the liver. In accordance with this, we noted an increase in the liver weight but no significant inflammation or fibrosis in 24abNOD control mice treated with Paquinimod for 4 weeks (S5 Fig).

**Fig 4:**
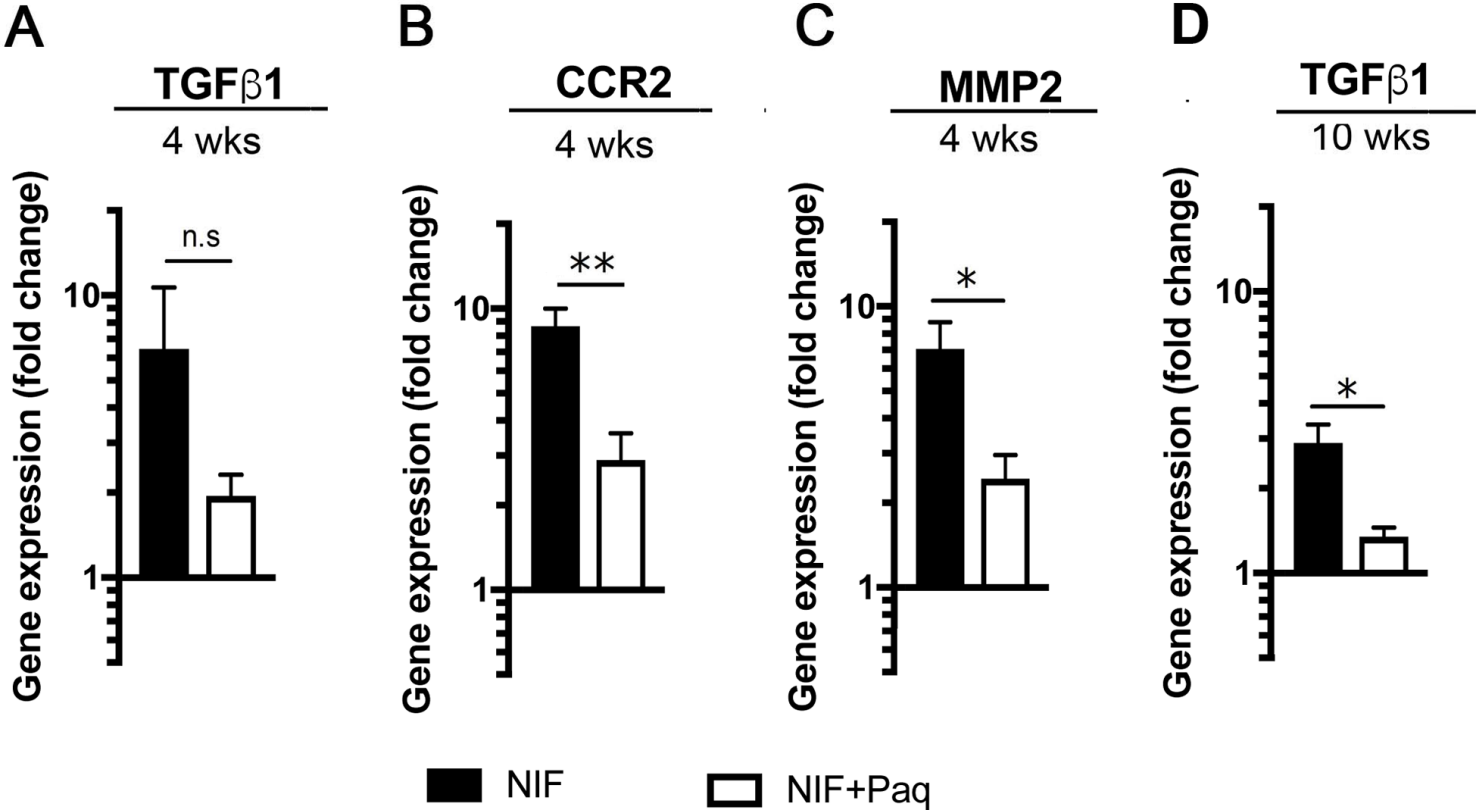
Paq treatment reduces gene expression of TGF-β, CCR2 and MMP2. Quantitative PCR analysis of gene expression in total liver lysate demonstrating a change in mRNA of (A) TGF-β, (B) CCR2 and (C) MMP2 in N-IF mice treated with Paquinimod (n = 3) or with vehicle (n = 4) for 4 weeks relative to 24αβNOD mice treated with vehicle for 4 weeks (n = 4). (D) Change in mRNA of TGF-β in N-IF mice treated with Paquinimod (n = 4) or with vehicle (n = 4) for 10 weeks relative to 24αβNOD mice (n = 4). The graphs show box plots and the horizontal line represents median value. Pooled data from two independent experiments are shown. n.s. = not significant; *P<0.05, **P<0.01, ***P<0.001, ****P<0.0001, unpaired t-test.

To further analyze the effect of Paquinimod treatment on the development of liver fibrosis, we next analyzed the expression of genes for transforming growth factor (TGF)-β, C-C chemokine receptor type 2 (CCR2) and matrix metalloproteinase (MMP) 2 all of which are associated with cells involved in the development of fibrosis [10–12]. Consistent with the accumulation of CD11b^+^ cells in the liver of the N-IF mice, we found an increased expression of TGF-β (Fig 4A) and an increased steady-state level of CCR2 (Fig 4B), and MMP2 (Fig 4C) mRNAs in the liver lysates from the N-IF mice relative to those from the 24αβNOD controls. A significant reduction in the expression of the CCR2, and MMP2 genes was observed in N-IF mice after Paquinimod treatment for 4 weeks (Fig 4B and 4C) while a reduction in the expression of TGF-β was only seen after continuing the treatment for 10 weeks (Fig 4A).

### Paquinimod treatment of N-IF mice leads to reduction of NKT cells and alters their cytokine profile

**Fig 5:**
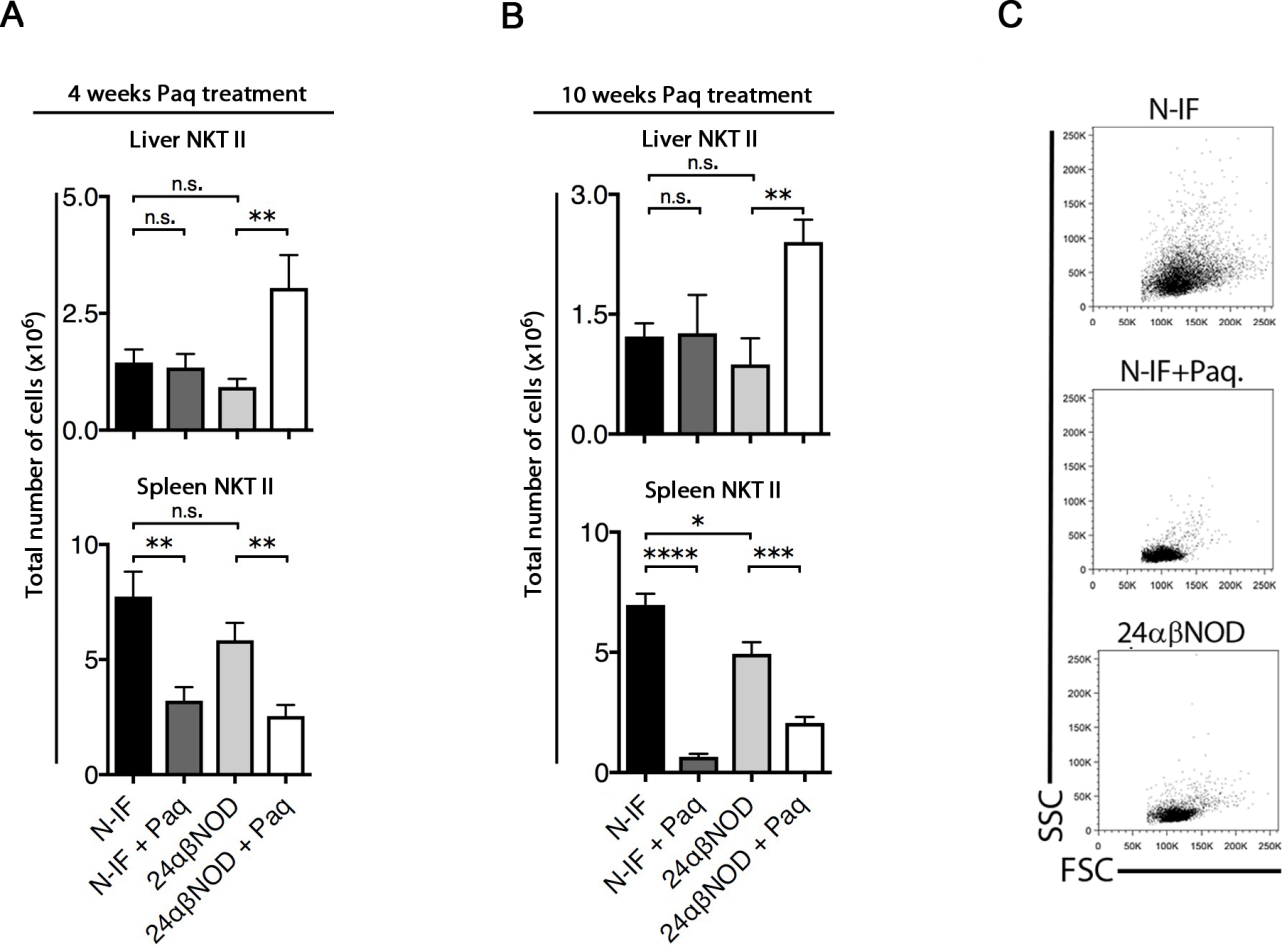
Paquinimod reduces the number and activation of NKT-II cells in N-IF mice. (A) Single cell suspensions from liver and spleen, analyzed by flow cytometry, from non-treated N-IF mice (n = 7), N-IF mice treated with Paquinimod for 4 weeks (n = 10), non-treated 24αβNOD mice (n = 8) and 24αβNOD mice treated with Paquinimod for 4 weeks (n = 7). (B) Non-treated N-IF mice (n = 5), N-IF mice treated with Paquinimod for 10 weeks (n = 8), non-treated 24αβNOD mice (n = 5) and 24αβNOD mice treated with Paquinimod for 10 weeks (n = 7). Total number of liver NKT-II cells is indicated. Number of NKT-II cells (Vα3.2^+^ Vβ9^+^) in liver (upper) and spleen (lower). Representative FSC/SSC plots displaying cells from the NKT-II gate (C). Results are from three pooled experiments (C). n.s. = not significant, *P<0.05, **P<0.01, ***P<0.001, ****P<0.0001, unpaired **t**-test.

Previous research has demonstrated that the transfer of total splenocytes, but not splenocytes depleted of NKT-II cells, from N-IF mice to NOD.Rag2^-/-^ recipients induces a N-IF phenotype in the recipients [13]. This finding suggests that the transgenic type II NKT (NKT-II, Vα3.2^+^ Vβ9^+^) cells promote the disease development in these mice. Therefore, we next investigated how Paquinimod treatment affected the NKT-II cells. As illustrated in Fig 5, treatment with Paquinimod significantly reduced the number of NKT-II cells in the spleen but not in the liver of N-IF mice (Fig 5). In fact, the proportion of NKT-II cells increased in the liver of treated N-IF mice (S6 Fig). When splenic NKT-II cells were analyzed further based on their FSC/SSC profile, an increase in SSC was detected in NKT-II cells from N-IF mice compared to 24αβNOD controls, suggesting an increased granularity and thus activation of these cells (Fig 5C). Paquinimod treatment reduced the SSC^hi^ profile of the NKT-II cells already after 4 weeks of treatment. This suggested a possible effect of Paquinimod on the functionality of these cells.

**Fig 6:**
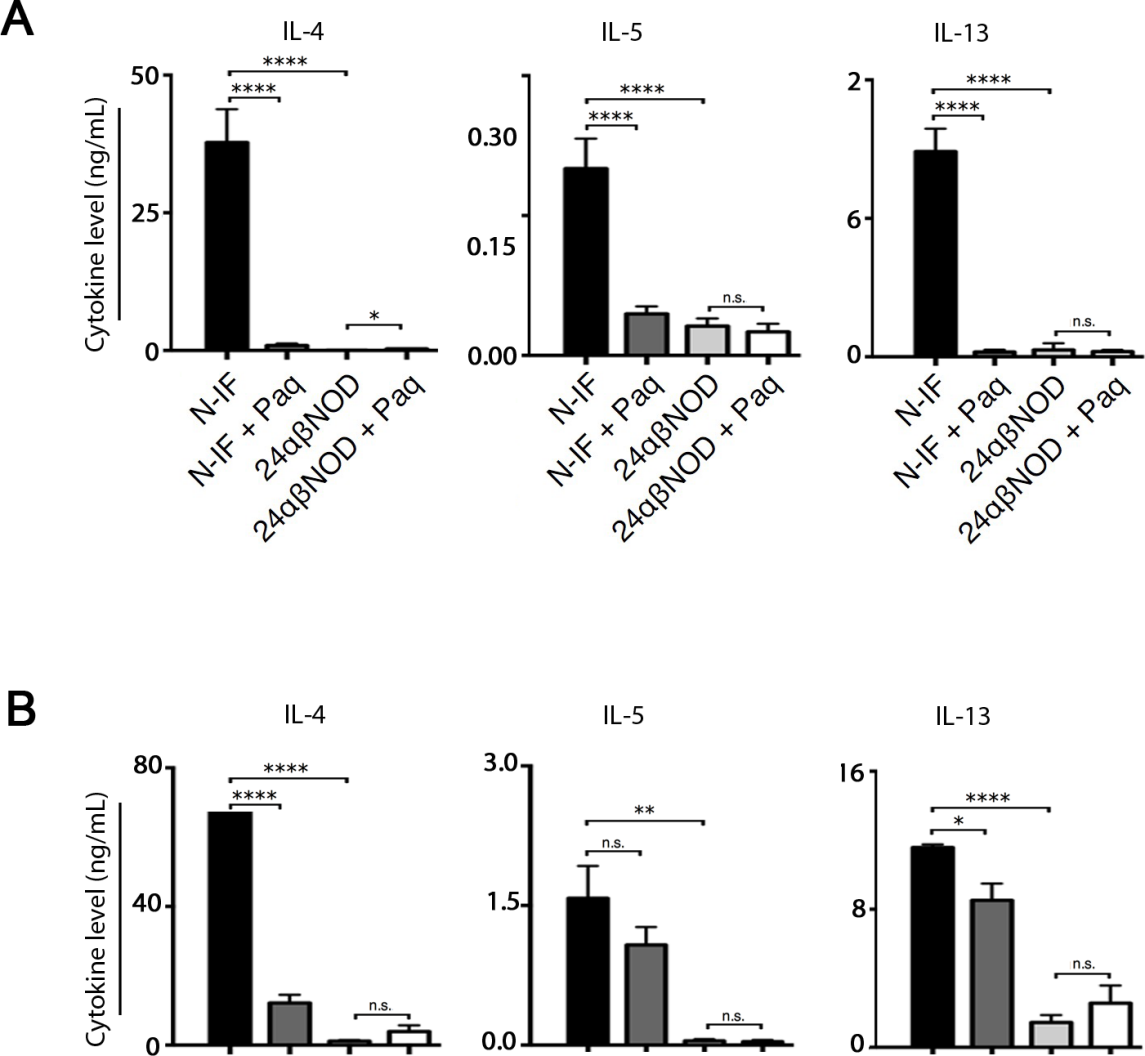
Paquinimod treatment reverses cytokine profile. IL-4, IL-5 and IL-13 levels in supernatants from (A) splenocytes or (B) total liver leukocytes from non-treated N-IF mice (n = 10), N-IF mice treated with Paquinimod for 4 weeks (n = 14), non-treated 24αβNOD mice (n = 13) and 24αβNOD mice treated with Paquinimod for 4 weeks (n = 12). Isolated cells were cultured for 24 h with anti-CD3 (4 μg/ml) activation. The results are from three pooled experiments. n.s. = not significant; *P<0.05, **P<0.01, ****P<0.0001, unpaired *t*-test.

We have previously reported that the N-IF mouse displays a mixed type 1/type 2-cytokine profile systemically as well as locally in the affected liver [8]. To test whether Paquinimod would affect cytokine production, total splenocytes from control and Paquinimod-treated N-IF and 24αβNOD control mice were activated with anti-CD3 antibodies *in vitro*. Following 24 h of activation, culture supernatants were collected, and the levels of various type 2 cytokines were determined. While supernatants of cells from N-IF mice contained high levels of type 2 cytokines such as IL-4, IL-5 and IL-13, supernatants of cells from N-IF mice treated with Paquinimod for 4 weeks had dramatically reduced levels of these cytokines (Fig 6A). Similar results were obtained when the experiment was performed using total liver leukocytes (Fig 6B). In conclusion, Paquinimod not only reduces the number of NKT-II cells in N-IF mice, but also their production of various type 2 cytokines.

### Paquinimod reduces the number of CD11b^+^ F4/80^+^ CD206^+^ macrophages and CD115^+^ Ly6C^hi^ cells in N-IF mice

**Fig 7:**
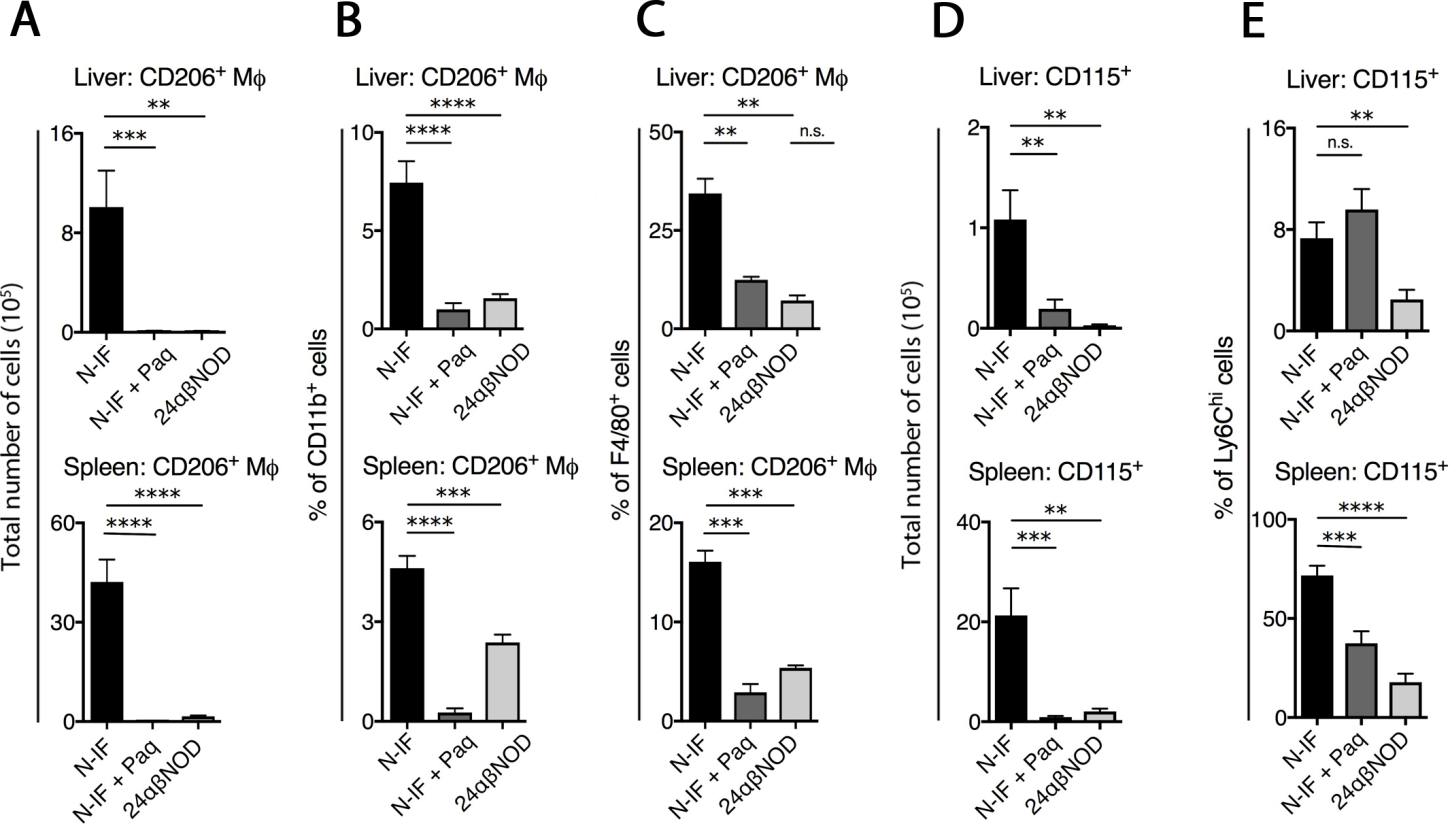
Paquinimod reduces CD115^+^ Ly6C^hi^ monocytes and CD115^+^ Ly6C^hi^ in N-IF mice. Single cell suspensions from the liver analyzed by flow cytometry, from non-treated N-IF mice (n = 7), N-IF mice treated with Paquinimod for 4 weeks (n = 10) and non-treated 24αβNOD mice (n = 8). (A) Total number and (B) frequency of CD206^+^ cells gated from CD11b^+^ cells; (C) frequency CD206^+^ cells gated from CD11b^+^ F4/80^+^ cells; (D) total number of CD115^+^ cells and (E) frequency of CD115^+^ cells gated from CD11b^+^Ly6C^hi^ cells. The pooled data from two independent experiments is shown. n.s. = not significant; *P<0.05, **P<0.01, ***P<0.001, ****P<0.0001, unpaired **t**-test.

Because Paquinimod was found to reduce the production of pro-fibrotic cytokines as well as liver fibrosis of the N-IF mice, we next evaluated the effect of this compound on cell populations known to contribute to fibrosis. It has previously been demonstrated that macrophages with an M2-like phenotype identified as CD11b^+^ F4/80^+^ CD206^+^ can promote the development of fibrosis through a TGF-β-mediated influence on fibroblast function [14–16]. Consistent with this finding, there was a dramatic increase both in the absolute number (Fig 7A) and frequency (Fig 7B) of CD11b^+^ F4/80^+^ CD206^+^ macrophages in both the liver (upper) and spleen (lower) of the N-IF mice compared to that in the 24αβNOD control mice. Four weeks of Paquinimod treatment completely reduced the accumulation of these cells in both compartments. In addition, Paquinimod reduced the frequency of CD206^+^ cells within the CD11b^+^ F4/80^+^ population in treated N-IF mice, suggesting that this compound also affected the polarization of macrophages (Fig 7C).

It has previously been shown that a CD115^+^ (colony-stimulating factor 1 receptor, CSF-1R^+^) subpopulation of Ly6C^hi^ monocytes is able to differentiate to M2-like macrophages [17, 18]. As expected, CD115^+^ Ly6C^hi^ monocytes were found to be increased both in number (Fig 7D) and frequency (Fig 7E) in both the liver (upper) and spleen (lower) of the N-IF mice relative to those in the 24αβNOD controls. In agreement with previous reports that Paquinimod can reduce this specific monocyte population [2], treatment of N-IF mice for 4 weeks reduced the number and frequency of this cell population in the spleen (Fig 7D and 7E). While Paquinimod treatment also reduced the number of CD115^+^ cells in the liver, their frequency within the Ly6C^hi^ population was not affected.

## Discussion

The N-IF mouse is a novel model for chronic liver inflammation and fibrosis driven by NKT-II cells producing a mixed type1/type 2-cytokine profile [8]. We report here that the treatment of N-IF mice, with the Q compound Paquinimod significantly reduced both the inflammation and the development of fibrosis in the liver. Because treatment was initiated at an age when the N-IF mice already had established inflammation and fibrosis in the liver, we conclude that Paquinimod treatment in this setting has a therapeutic effect.

In untreated N-IF mice, the transgenic NKT-II cells in the liver as well as in the spleen produce high levels of type 2 cytokines such as IL-4, IL-5, and IL-13 as well as pro-inflammatory cytokines like IFN-γ, IL-6 and IL-2. This mixed cytokine profile is likely a causative factor in the inflammation and fibrosis observed in the N-IF mouse [8]. In Paquinimod-treated N-IF mice the reversion of liver inflammation and fibrosis coincided with a reduced production of the pro-inflammatory cytokines and the Th2 cytokines both in the spleen and the liver. This suggests that the disease-inhibiting effect of Paquinimod was, at least in part, mediated by modifying the NKT-II cells in the N-IF mouse. In support of this, we noted a reduction in the size and granularity of the NKT-II cells after Paquinimod treatment suggesting that these cells were less activated after treatment. We also observed a reduction in the absolute number of the NKT-II cells in the spleen but not in the liver of the treated N-IF mice. The relative loss of this cell population in the spleen supports the notion that Paquinimod directly inhibits the NKT cell population. A plausible explanation for the absence of a corresponding reduction in the liver may be that a larger proportion of the remaining NKT cells preferentially are recruited to the liver in line with the well established preferred homing of NKT cells to this organ [19, 20].

Macrophages play an important role in fibrogenesis as well as in fibrolysis [14, 15]. Accordingly, N-IF mice displayed increased numbers of CD11b^+^ F4/80^+^ CD206^+^ macrophages in the liver and spleen. In addition to the observed effect on NKT-II cells, this macrophage population was significantly reduced in liver and spleen of N-IF mice. Moreover, Paquinimod treatment caused a phenotypic change in the CD11b^+^ F4/80^+^ macrophage population. As expected, this alteration in macrophage polarization coincided with a reduction in the frequency of Ly6C^hi^ monocytes and SiglecF^+^ eosinophils and an increased frequency of Ly6G^hi^ neutrophils. These data concur with previously reported data obtained in a model of peritonitis [2], as well as in tumor models treated with Tasquinimod, another Q compound [21]. The liver weight in Paquinimod-treated N-IF mice was not significantly affected despite the reduction in inflammation and fibrosis. The observation that Paquinimod increased the liver weight in the 24αβNOD control mice could partly explain this unexpected finding. This effect of Paquinimod on the liver of treated 24αβNOD control mice is likely to be the result of the increase in liver metabolism previously reported.

In summary, this study provides evidence that the Q substance, Paquinimod, previously recognized as an anti-inflammatory agent and an orphan drug for the treatment of scleroderma, constitutes a more general drug candidate for the treatment of fibrotic conditions with the potential to reverse already established fibrosis.

## Supporting information

S1 Fig Gating strategy. Gating strategy for splenic: (A, B) NKT cells (C, D) monocytes/granulocytes, (E, F) mast cells/ basophils / macrophages in N-IF mice (A, C, E) and in 24αβNOD mice (B, D, F)
(PDF)

S2 Fig Composition of subsets of CD11b^+^ cells in the liver. Pie charts showing the frequency of Ly6C^hi^, Ly6G^hi^ and SiglecF^+^ cells among CD11b+ liver cells isolated from N-IF mice (n = 4), N-IF mice treated with Paquinimod for 4 weeks (n = 6) and 24αβNOD mice (n = 5). Representative results of two independent experiments are shown.
(PDF)

S3 Fig Effect of Paquinimod treatment for 10 weeks on cytokine expression. TNFα, IL-6 and IL-10 levels in supernatants from total liver leukocytes from non-treated N-IF mice (n = 6), N-IF mice treated with Paquinimod for 10 weeks (n = 6), non-treated 24αβNOD mice (n = 5) and 24αβNOD mice treated with Paquinimod for 10 weeks (n = 8). Isolated cells were cultured for 24 h with anti-CD3 (4 μg/ml) activation. The results are from two pooled experiments. n.s. = not significant; *P<0.05, **P<0.01, ****P<0.0001, unpaired *t*-test.
(PDF)

S4 Fig Histology and quantification of hydroxyproline content in the liver after 10 weeks treatment with Paquinimod. (A). Representative Masson’s trichrome-stained section of liver from N-IF mouse treated with Paquinimod for 10 weeks. (B) Quantification of collagen in the liver of N-IF mice after 10 weeks of Paquinimod treatment (n = 8) and of age matched untreated N-IF mice (n = 5) or 24αβNOD mice (n = 5), based on hydroxyproline measurements. Representative results of two independent experiments are shown. n.s. = not significant, *P<0.05, **P<0.01, ***P<0.001, ****P<0.0001, unpaired **t**-test.
(PDF)

S5 Fig Liver weight and histology of 24αβNOD control mice treated with Paquinimod for 4 weeks. (A). Liver weight and (B) representative Masson’s trichrome-stained section of liver from 24αβNOD mice non-treated (n = 9) or treated with Paquinimod for 4 weeks (n = 8). n.s. = not significant, *P<0.05, **P<0.01, ***P<0.001, ****P<0.0001, unpaired **t**-test.
(PDF)

S6 Fig Paquinimod alters the frequency of NKT-II cells in N-IF mice. Single cell suspensions from liver and spleen, analyzed by flow cytometry, from (A) non-treated N-IF mice (n = 7), N-IF mice treated with Paquinimod for 4 weeks (n = 6), non-treated 24αβNOD mice (n = 8) and 24αβNOD mice treated with Paquinimod for 4 weeks (n = 7) and (B) from non-treated N-IF mice (n = 5), N-IF mice treated with Paquinimod (n = 8), non-treated 24αβNOD mice (n = 5) and 24αβNOD mice treated with Paquinimod for 10 weeks (n = 8). Frequency of NKT-II cells gated from viable CD45^+^ cells in liver (upper) and spleen (lower). Results are from two pooled experiments (C). n.s. = not significant, *P<0.05, **P<0.01, ****P<0.0001 Unpaired *t*-test.
(PDF)

## References

[1] Schelbergen RF, Geven EJ, van den Bosch MH, Eriksson H, Leanderson T, Vogl T, et al Prophylactic treatment with S100A9 inhibitor paquinimod reduces pathology in experimental collagenase-induced osteoarthritis. Ann Rheum Dis. 2015;74(12):2254–8. 10.1136/annrheumdis-2014-206517 . 25969431

[2] Deronic A, Helmersson S, Leanderson T, Ivars F. The quinoline-3-carboxamide paquinimod (ABR-215757) reduces leukocyte recruitment during sterile inflammation: Leukocyte- and context-specific effects. International immunopharmacology. 2014;18(2):290–7. 10.1016/j.intimp.2013.12.008 . 24370393

[3] Bengtsson AA, Sturfelt G, Lood C, Ronnblom L, van Vollenhoven RF, Axelsson B, et al Pharmacokinetics, tolerability, and preliminary efficacy of paquinimod (ABR-215757), a new quinoline-3-carboxamide derivative: Studies in lupus-prone mice and a multicenter, randomized, double-blind, placebo-controlled, repeat-dose, dose-ranging study in patients with systemic lupus erythematosus. Arthritis and rheumatism. 2012;64(5):1579–88. 10.1002/art.33493 . 22131101

[4] Hesselstrand R, Distler JH, Riemekasten G, Törngren M, Nyhlén HC, Stenström M, et al An open-label study to evaluate biomarkers and safety in systemic sclerosis (SSc) patients treated with paquinimod (ABR-215757). Ann Rheum Dis. 2014;73 (Suppl2):566–7. 10.1136/annrheumdis-2014-eular.4207

[5] Bjork P, Bjork A, Vogl T, Stenstrom M, Liberg D, Olsson A, et al Identification of human S100A9 as a novel target for treatment of autoimmune disease via binding to quinoline-3-carboxamides. PLoS biology. 2009;7(4):e97 10.1371/journal.pbio.1000097 ; PubMed Central PMCID: PMCPMC2671563. 19402754 PMC2671563

[6] Sinha P, Okoro C, Foell D, Freeze HH, Ostrand-Rosenberg S, Srikrishna G. Proinflammatory S100 proteins regulate the accumulation of myeloid-derived suppressor cells. Journal of immunology. 2008;181(7):4666–75. ; PubMed Central PMCID: PMCPMC2810501. 18802069 10.4049/jimmunol.181.7.4666 PMC2810501

[7] Cheng P, Corzo CA, Luetteke N, Yu B, Nagaraj S, Bui MM, et al Inhibition of dendritic cell differentiation and accumulation of myeloid-derived suppressor cells in cancer is regulated by S100A9 protein. The Journal of experimental medicine. 2008;205(10):2235–49. 10.1084/jem.20080132 ; PubMed Central PMCID: PMCPMC2556797. 18809714 PMC2556797

[8] Fransen-Pettersson N, Duarte N, Nilsson J, Lundholm M, Mayans S, Larefalk A, et al A New Mouse Model That Spontaneously Develops Chronic Liver Inflammation and Fibrosis. PLoS One. 2016;11(7):e0159850 Epub 2016/07/22. 10.1371/journal.pone.0159850 ; PubMed Central PMCID: PMCPMC4956255. 27441847 PMC4956255

[9] Kaye J, Piryatinsky V, Birnberg T, Hingaly T, Raymond E, Kashi R, et al Laquinimod arrests experimental autoimmune encephalomyelitis by activating the aryl hydrocarbon receptor. Proc Natl Acad Sci U S A. 2016;113(41):E6145–E52. Epub 2016/09/28. 10.1073/pnas.1607843113 ; PubMed Central PMCID: PMCPMC5068259. 27671624 PMC5068259

[10] Baeck C, Wei X, Bartneck M, Fech V, Heymann F, Gassler N, et al Pharmacological inhibition of the chemokine C-C motif chemokine ligand 2 (monocyte chemoattractant protein 1) accelerates liver fibrosis regression by suppressing Ly-6C(+) macrophage infiltration in mice. Hepatology. 2014;59(3):1060–72. 10.1002/hep.26783 . 24481979

[11] Giannandrea M, Parks WC. Diverse functions of matrix metalloproteinases during fibrosis. Disease models & mechanisms. 2014;7(2):193–203. 10.1242/dmm.012062 ; PubMed Central PMCID: PMCPMC3917240. 24713275 PMC3917240

[12] Seki E, de Minicis S, Inokuchi S, Taura K, Miyai K, van Rooijen N, et al CCR2 promotes hepatic fibrosis in mice. Hepatology. 2009;50(1):185–97. 10.1002/hep.22952 ; PubMed Central PMCID: PMCPMC2705470. 19441102 PMC2705470

[13] Fransén-Pettersson N, Duarte, Lundholm, Mayans, Larefalk, Hannibal, et al A new mouse model for primary biliary cirrhosis that spontaneously develops biliary disease with liver fibrosis. Submitted to J Immunol.

[14] Lech M, Anders HJ. Macrophages and fibrosis: How resident and infiltrating mononuclear phagocytes orchestrate all phases of tissue injury and repair. Biochimica et biophysica acta. 2013;1832(7):989–97. 10.1016/j.bbadis.2012.12.001 . 23246690

[15] Murray PJ, Wynn TA. Protective and pathogenic functions of macrophage subsets. Nature reviews Immunology. 2011;11(11):723–37. 10.1038/nri3073 ; PubMed Central PMCID: PMCPMC3422549. 21997792 PMC3422549

[16] Karlmark KR, Weiskirchen R, Zimmermann HW, Gassler N, Ginhoux F, Weber C, et al Hepatic recruitment of the inflammatory Gr1+ monocyte subset upon liver injury promotes hepatic fibrosis. Hepatology. 2009;50(1):261–74. 10.1002/hep.22950 . 19554540

[17] Auffray C, Sieweke MH, Geissmann F. Blood monocytes: Development, heterogeneity, and relationship with dendritic cells. Annual review of immunology. 2009;27:669–92. 10.1146/annurev.immunol.021908.132557 . 19132917

[18] Hume DA, MacDonald KP. Therapeutic applications of macrophage colony-stimulating factor-1 (CSF-1) and antagonists of CSF-1 receptor (CSF-1R) signaling. Blood. 2012;119(8):1810–20. 10.1182/blood-2011-09-379214 . 22186992

[19] Eberl G, Lees R, Smiley ST, Taniguchi M, Grusby MJ, MacDonald HR. Tissue-specific segregation of CD1d-dependent and CD1d-independent NK T cells. J Immunol. 1999;162(11):6410–9. Epub 1999/06/03. . 10352254

[20] Matsuda JL, Naidenko OV, Gapin L, Nakayama T, Taniguchi M, Wang CR, et al Tracking the response of natural killer T cells to a glycolipid antigen using CD1d tetramers. J Exp Med. 2000;192(5):741–54. Epub 2000/09/07. ; PubMed Central PMCID: PMCPMC2193268. 10974039 10.1084/jem.192.5.741 PMC2193268

[21] Shen L, Sundstedt A, Ciesielski M, Miles KM, Celander M, Adelaiye R, et al Tasquinimod modulates suppressive myeloid cells and enhances cancer immunotherapies in murine models. Cancer immunology research. 2015;3(2):136–48. 10.1158/2326-6066.CIR-14-0036 ; PubMed Central PMCID: PMCPMC4323929. 25370534 PMC4323929

