# Supplementary figures and images for: The immunomodulatory quinoline-3-carboxamide paquinimod reverses established fibrosis in a novel mouse model for liver fibrosis
Source: PLoS One. 2018 Sep 5;13(9):e0203228. doi: 10.1371/journal.pone.0203228 (PMC6124744; doi:10.1371/journal.pone.0203228)

S1A Fig

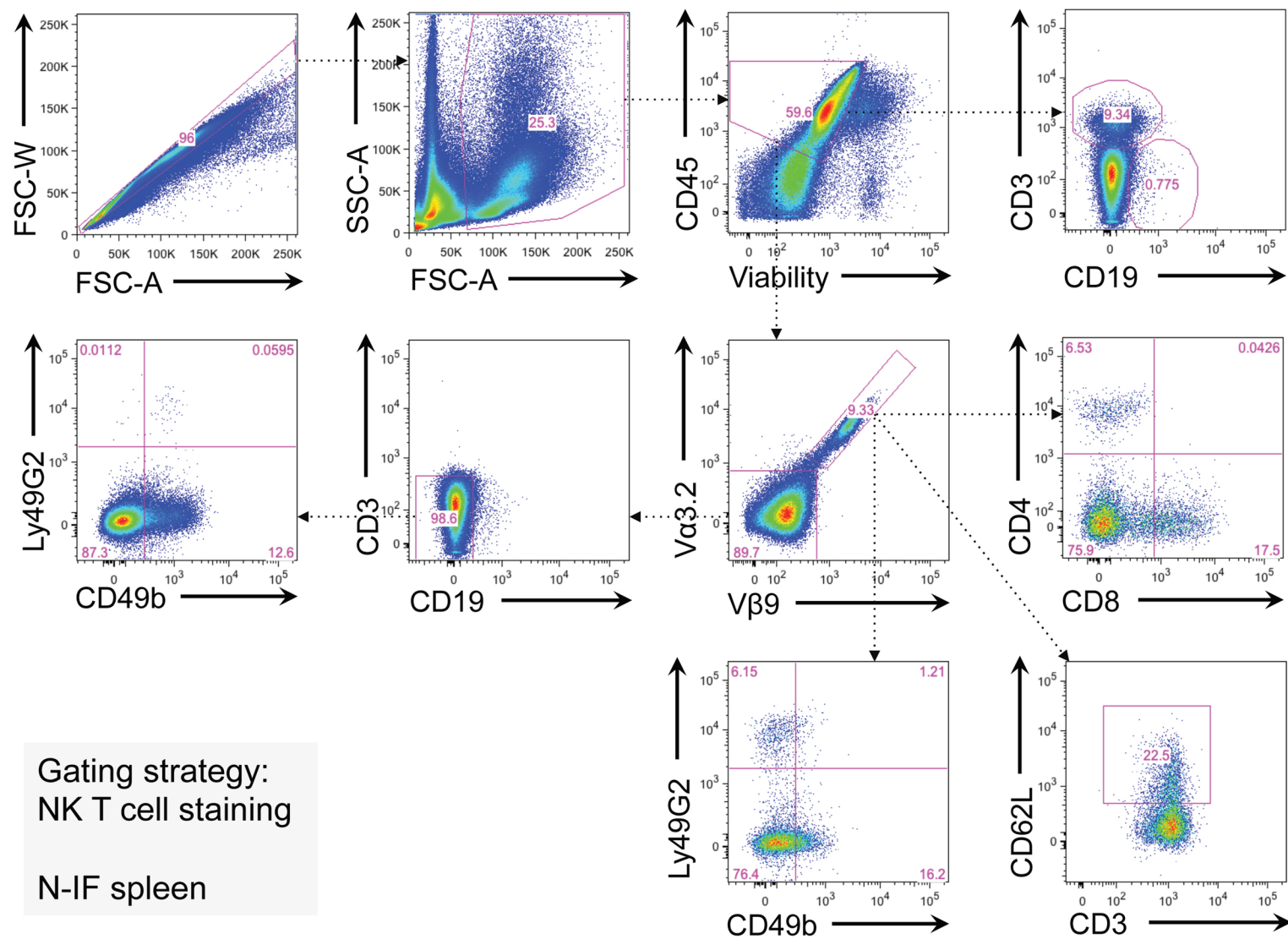

S1B Fig

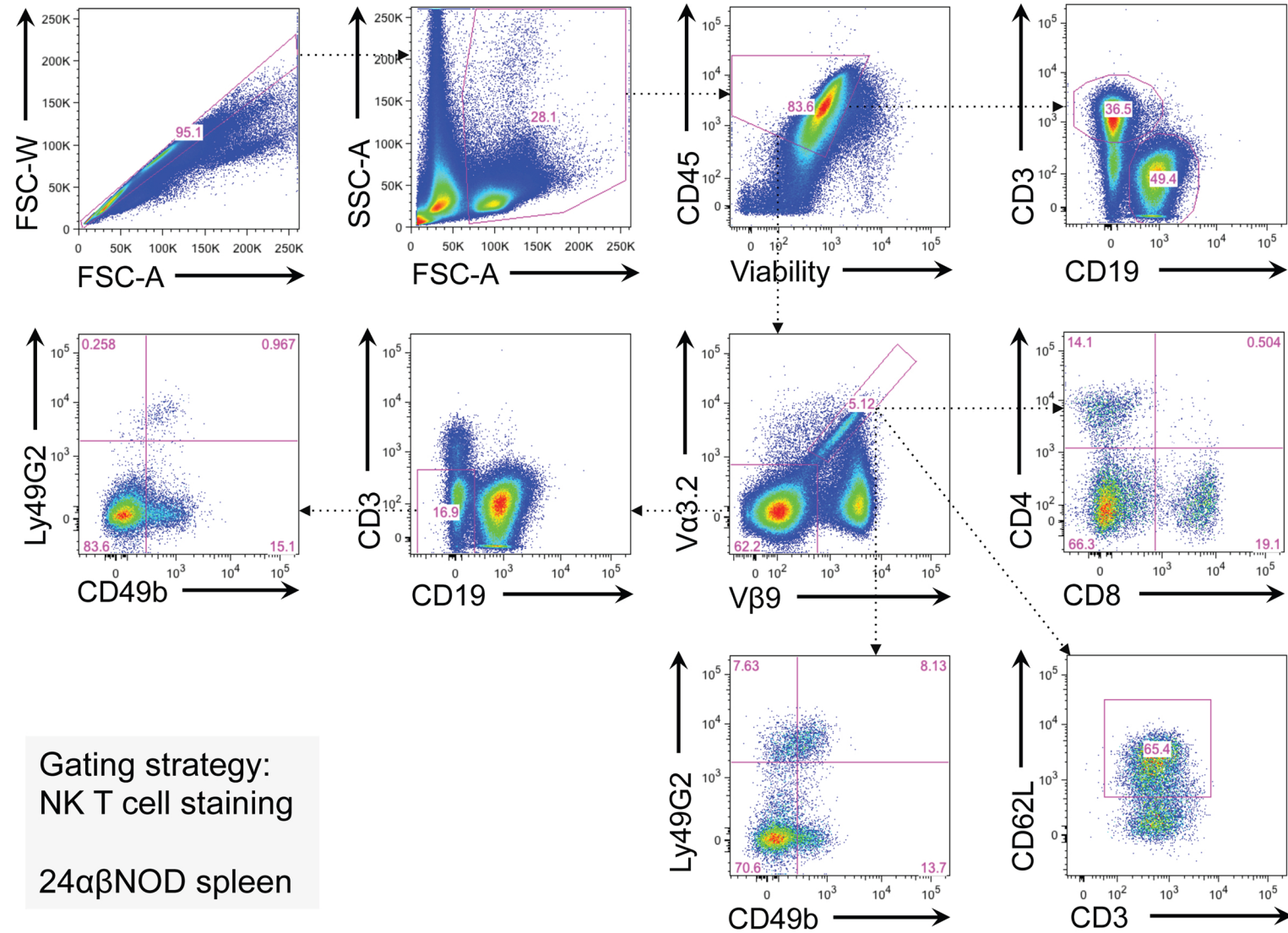

## S1C Fig

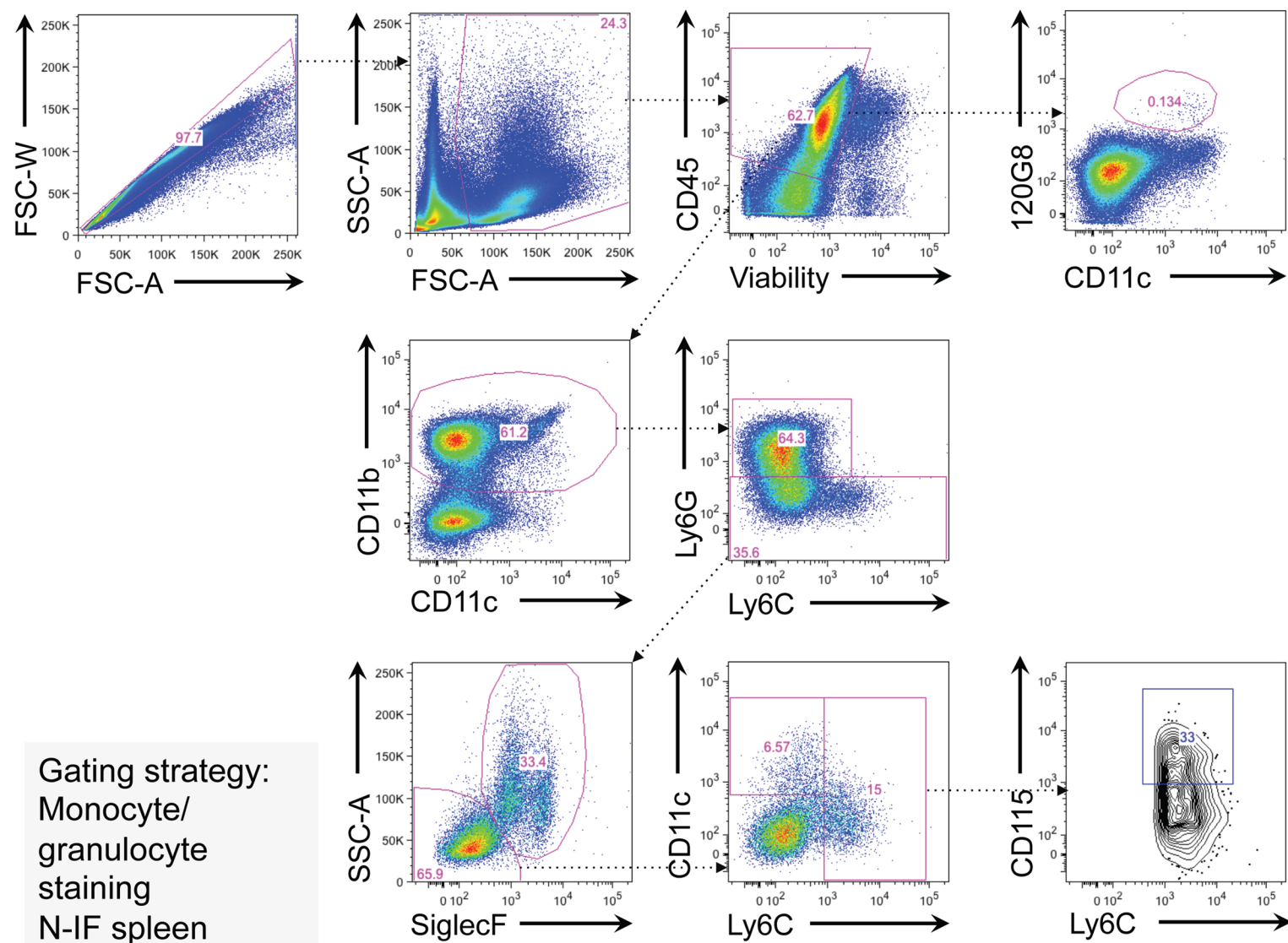

# S1D Fig

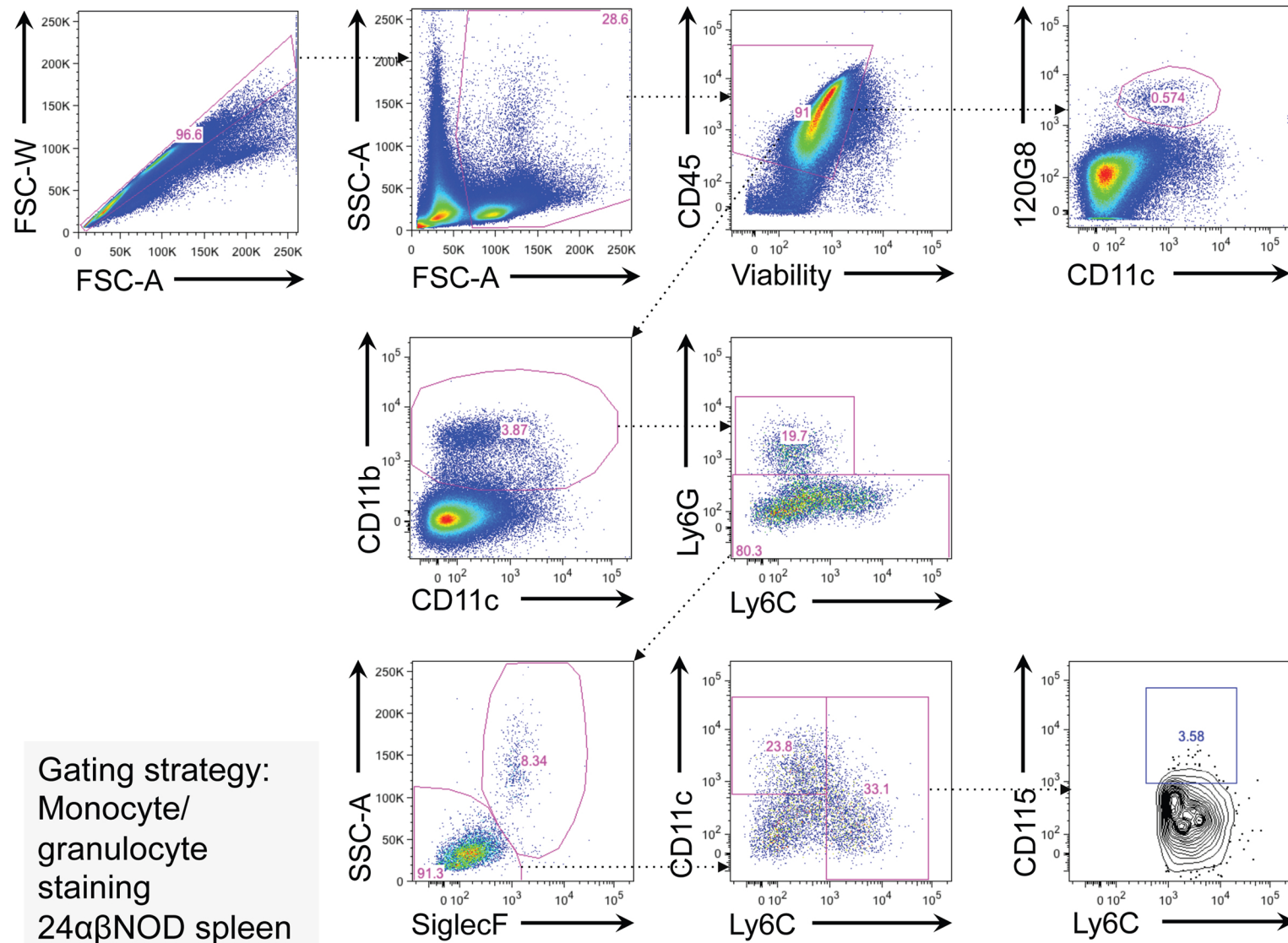

## S1E Fig

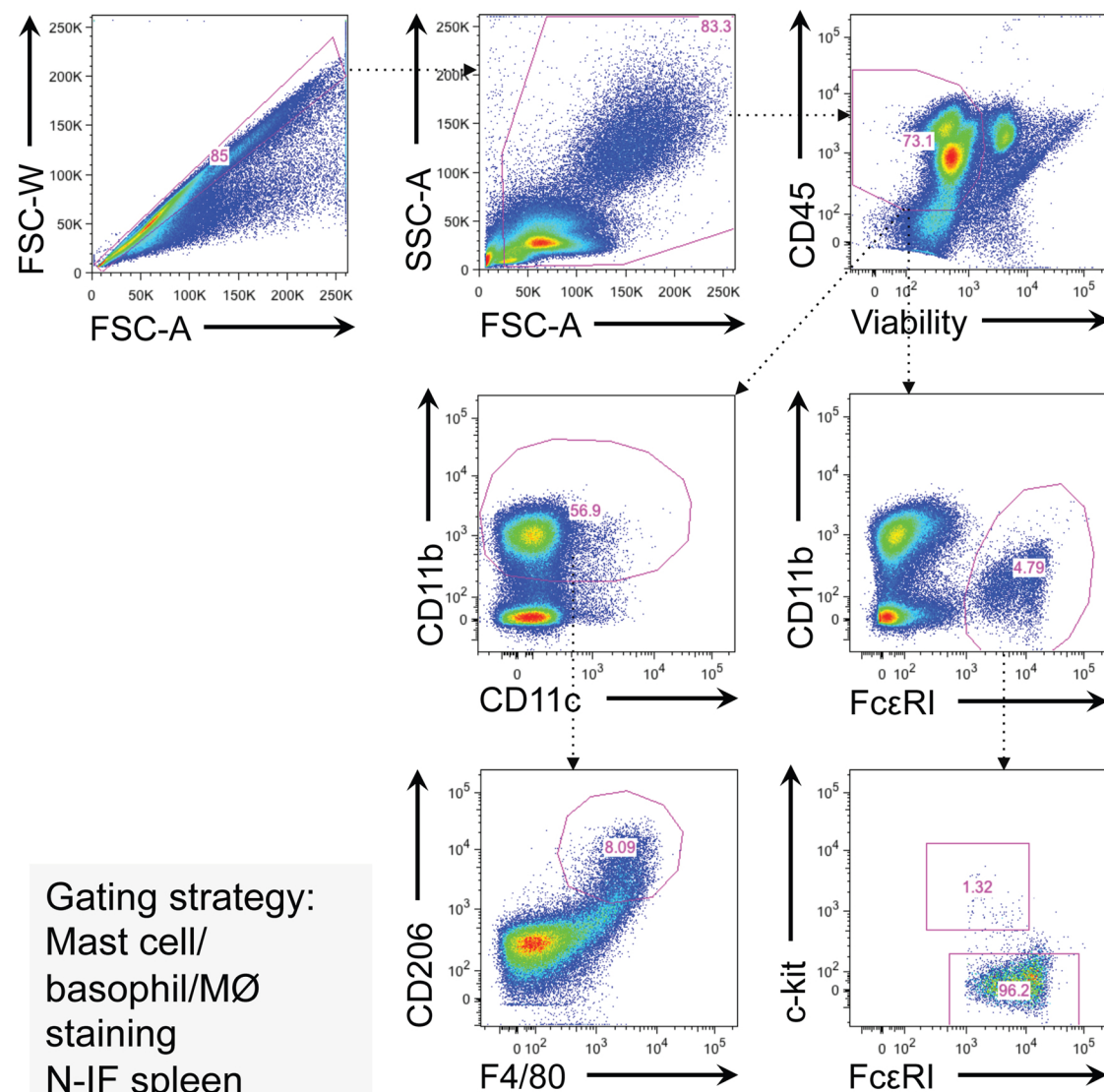

S1F Fig

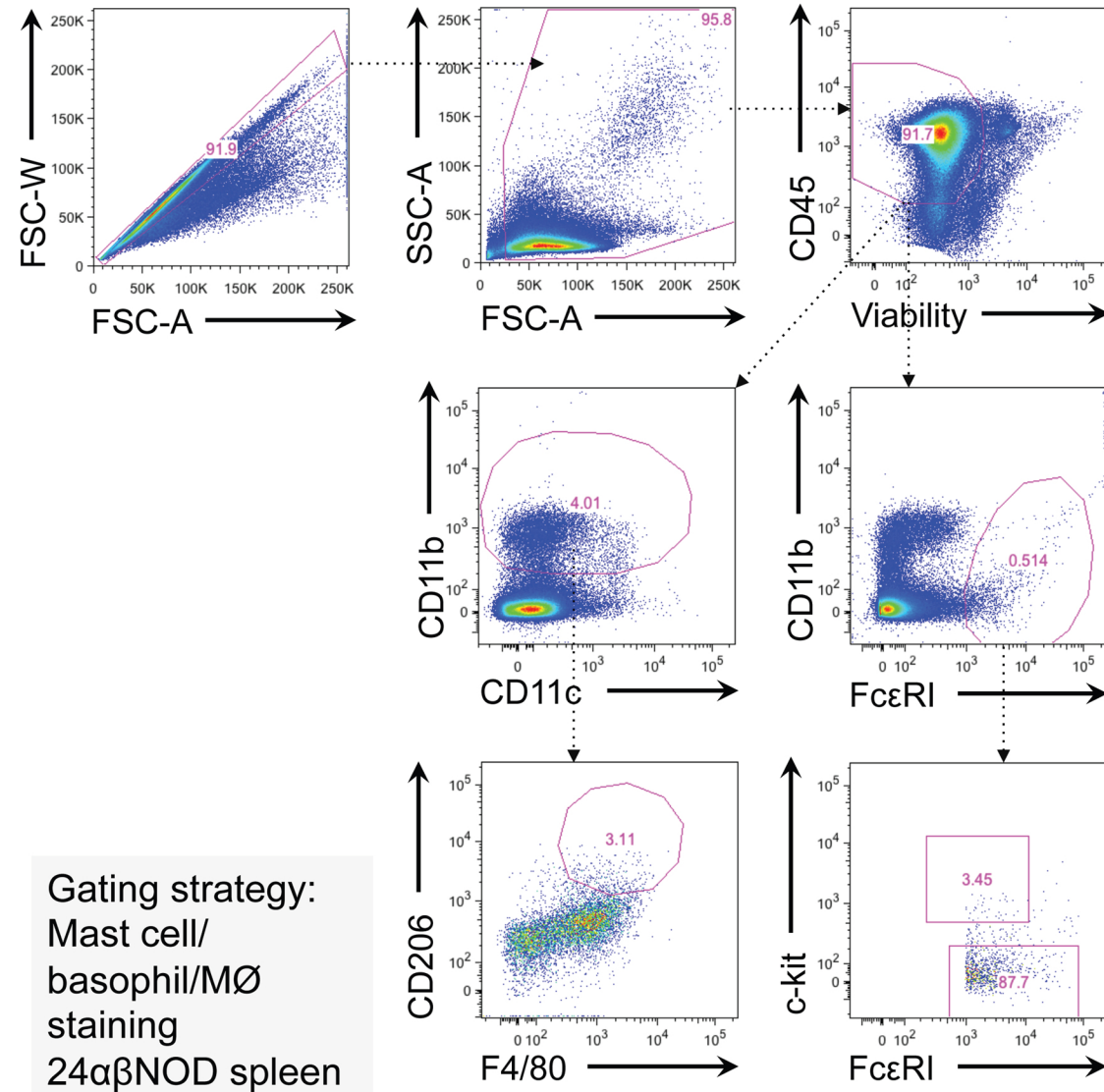

Supplement: S1 Fig — Gating strategy for splenic: (A, B) NKT cells (C, D) monocytes/granulocytes, (E, F) mast cells/ basophils / macrophages in N-IF mice (A, C, E) and in 24αβNOD mice (B, D, F) (PDF) [file pone.0203228.s001.pdf]

S. Figure 2

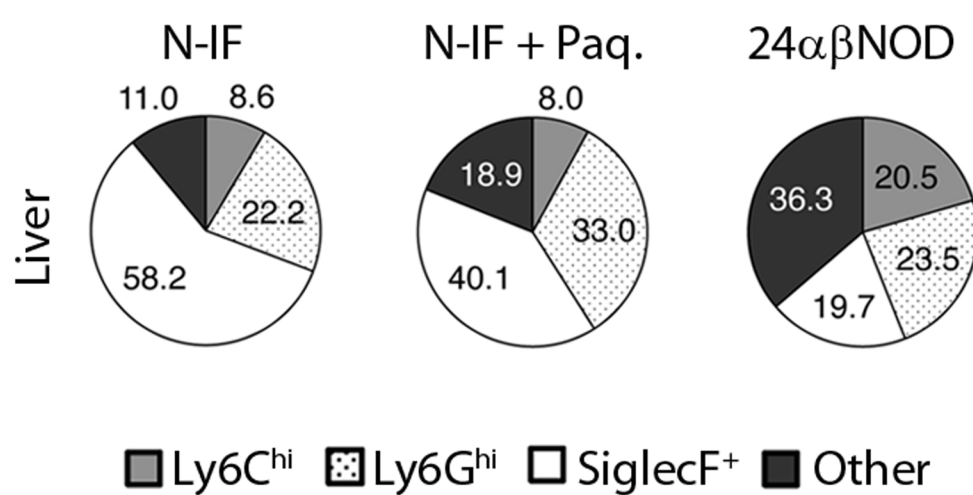

Supplement: S2 Fig — Pie charts showing the frequency of Ly6Chi, Ly6Ghi and SiglecF+ cells among CD11b+ liver cells isolated from N-IF mice (n = 4), N-IF mice treated with Paquinimod for 4 weeks (n = 6) and 24αβNOD mice (n = 5). Representative results of two independent experiments are shown. (PDF) [file pone.0203228.s002.pdf]

S3 Fig

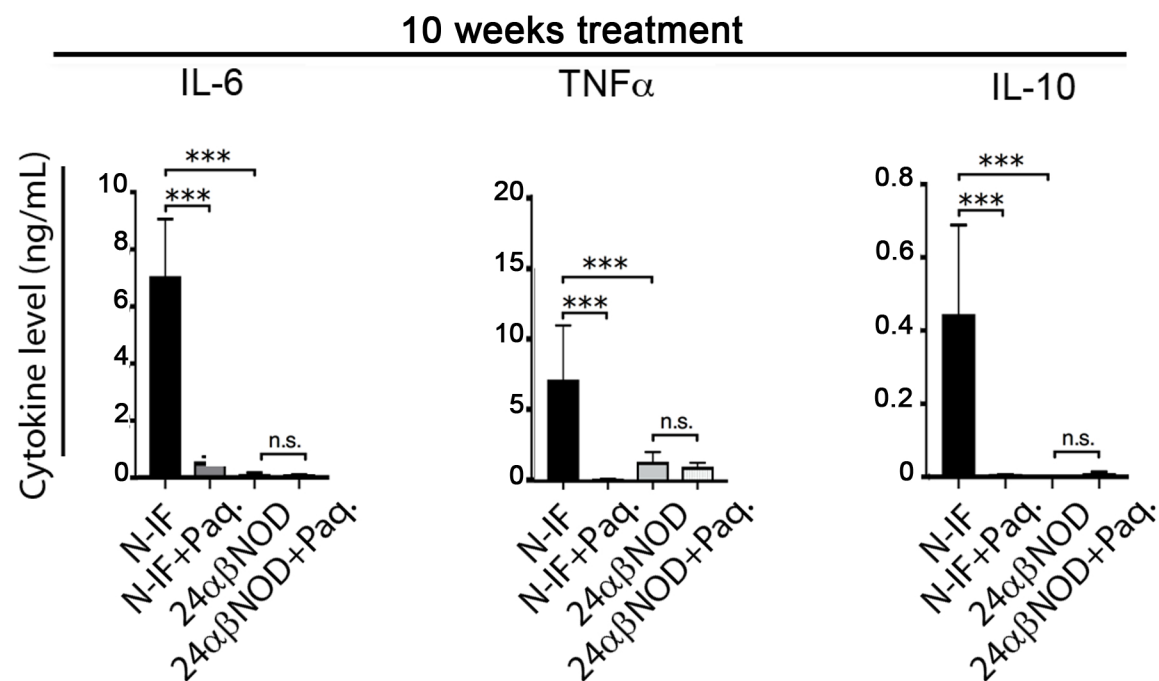

Supplement: S3 Fig — TNFα, IL-6 and IL-10 levels in supernatants from total liver leukocytes from non-treated N-IF mice (n = 6), N-IF mice treated with Paquinimod for 10 weeks (n = 6), non-treated 24αβNOD mice (n = 5) and 24αβNOD mice treated with Paquinimod for 10 weeks (n = 8). Isolated cells were cultured for 24 h with anti-CD3 (4 μg/ml) activation. The results are from two pooled experiments. n.s. = not significant; *P<0.05, **P<0.01, ****P<0.0001, unpaired t-test. (PDF) [file pone.0203228.s003.pdf]

S4 Fig

A

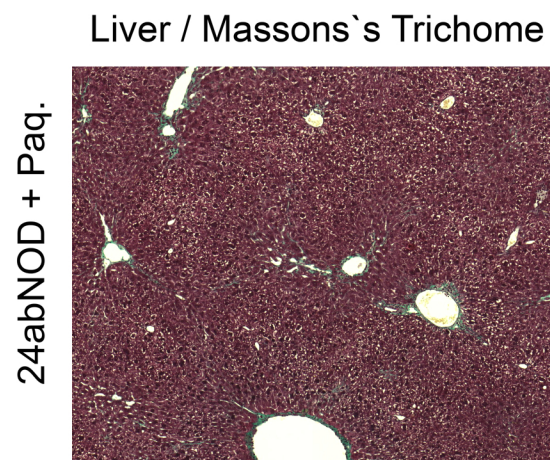

B

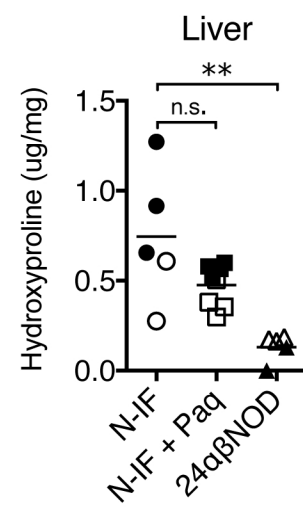

Supplement: S4 Fig — (A). Representative Masson’s trichrome-stained section of liver from N-IF mouse treated with Paquinimod for 10 weeks. (B) Quantification of collagen in the liver of N-IF mice after 10 weeks of Paquinimod treatment (n = 8) and of age matched untreated N-IF mice (n = 5) or 24αβNOD mice (n = 5), based on hydroxyproline measurements. Representative results of two independent experiments are shown. n.s. = not significant, *P<0.05, **P<0.01, ***P<0.001, ****P<0.0001, unpaired t-test. (PDF) [file pone.0203228.s004.pdf]

S5 Fig

A

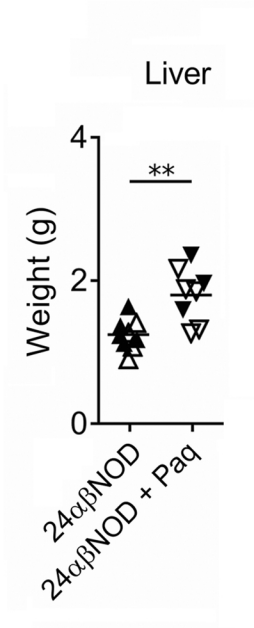

B

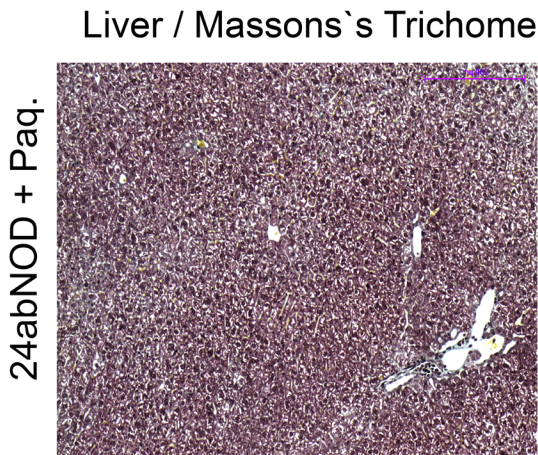

Supplement: S5 Fig — (A). Liver weight and (B) representative Masson’s trichrome-stained section of liver from 24αβNOD mice non-treated (n = 9) or treated with Paquinimod for 4 weeks (n = 8). n.s. = not significant, *P<0.05, **P<0.01, ***P<0.001, ****P<0.0001, unpaired t-test. (PDF) [file pone.0203228.s005.pdf]

S6 Fig

A

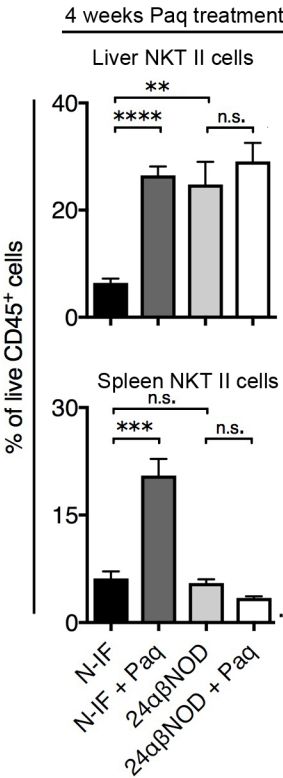

B

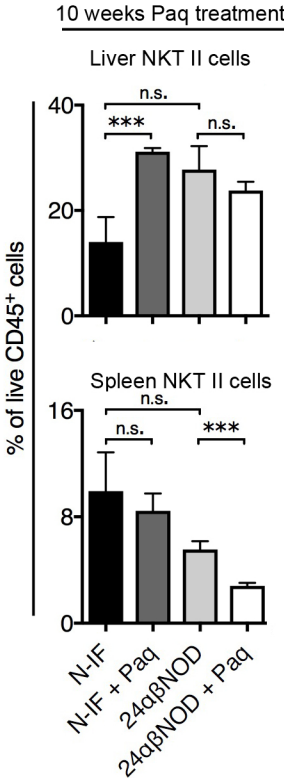

Supplement: S6 Fig — Single cell suspensions from liver and spleen, analyzed by flow cytometry, from (A) non-treated N-IF mice (n = 7), N-IF mice treated with Paquinimod for 4 weeks (n = 6), non-treated 24αβNOD mice (n = 8) and 24αβNOD mice treated with Paquinimod for 4 weeks (n = 7) and (B) from non-treated N-IF mice (n = 5), N-IF mice treated with Paquinimod (n = 8), non-treated 24αβNOD mice (n = 5) and 24αβNOD mice treated with Paquinimod for 10 weeks (n = 8). Frequency of NKT-II cells gated from viable CD45+ cells in liver (upper) and spleen (lower). Results are from two pooled experiments (C). n.s. = not significant, *P<0.05, **P<0.01, ****P<0.0001 Unpaired t-test. (PDF) [file pone.0203228.s006.pdf]
